# Elucidation of the genetic and epigenetic landscape alterations in RNA binding proteins in glioblastoma

**DOI:** 10.18632/oncotarget.14287

**Published:** 2016-12-27

**Authors:** Shruti Bhargava, Vikas Patil, Kulandaivelu Mahalingam, Kumaravel Somasundaram

**Affiliations:** ^1^ Department of Microbiology and Cell Biology, Indian Institute of Science, Bangalore-560012, India; ^2^ Department of Bio-Medical Sciences, School of Biosciences and Technology, VIT University, Vellore-632014, India

**Keywords:** glioblastoma, RNA binding proteins, regulation, signature, cancer stem cells

## Abstract

RNA binding proteins (RBPs) have been implicated in cancer development. An integrated bioinformatics analysis of RBPs (n = 1756) in various datasets (n = 11) revealed several genetic and epigenetically altered events among RBPs in glioblastoma (GBM). We identified 13 mutated and 472 differentially regulated RBPs in GBM samples. Mutations in AHNAK predicted poor prognosis. Copy number variation (CNV), DNA methylation and miRNA targeting contributed to RBP differential regulation. Two sets of differentially regulated RBPs that may be implicated in initial astrocytic transformation and glioma progression were identified. We have also identified a four RBP (NOL3, SUCLG1, HERC5 and AFF3) signature, having a unique expression pattern in glioma stem-like cells (GSCs), to be an independent poor prognostic indicator in GBM. RBP risk score derived from the signature also stratified GBM into low-risk and high-risk groups with significant survival difference. Silencing NOL3, SUCLG1 and HERC5 inhibited GSC maintenance. Gene set enrichment analysis of differentially regulated genes between high-risk and low-risk underscored the importance of inflammation, EMT and hypoxia in high-risk GBM. Thus, we provide a comprehensive overview of genetic and epigenetic regulation of RBPs in glioma development and progression.

## INTRODUCTION

Glioblastoma (GBM) is one of the most lethal primary brain tumors. In spite of several improvements in therapeutic modalities, the median survival remains low at 14-16 months [1]. Currently, the field of glioma research is focussed on developing tools for early detection, reliable prognostic and predictive biomarkers and novel therapies that can overcome the resistance.

Gene regulation in eukaryotes is a multi-step process. Nascent RNA formed after transcription generally undergoes modification, transport, localization and translation [2–4]. Extensive efforts in the past decade have gone in understanding few of these steps including transcription, splicing, and translation. With the advent of high throughput techniques in genomics, the focus of the research had been on changes in transcript levels due to genetic and epigenetic mechanisms. Numerous studies often indicate a lack of significant correlation between the transcript and protein levels in cells [5]. These observations led to the belief that additional processes may also play important role in affecting the cellular pool of proteins translated from their respective transcripts. This paradox can be further explained by the identification of post transcriptional regulatory check points which contribute immensely to the protein level regulation. These check points mainly consist of regulation mediated by non-coding RNAs (miRNAs and lncRNAs) and the RNA binding proteins [6].

RNA binding proteins (RBPs) partner the nascent RNA throughout its journey in the cell. The multi-functionality and the vast repertoire of targets regulated by RBPs make them important post transcriptional regulators. Thus, understanding of structure and function of this class of molecules becomes imperative to appreciate the multitude of processes altered by the mis-regulation of these proteins. Increasing volume of evidence prove that the RBPs are mis-regulated in diseased conditions including cancer. Many of these mis-regulated RBPs have also been shown to contribute to pathogenesis of cancer [3, 4, 7].

In the present study, we sought to understand the various aspects of RBP biology in glioblastoma (GBM). For this purpose, a catalogue of RBPs (till date known) was made using the datasets from existing literature. These RBPs were analysed for expression and sequence alterations in GBM by analysing the various publically available datasets. Further, we went on the quest to enlist the possible mechanisms which could contribute to the altered expression of these RBPs in GBM. We also analysed the expression of RBPs in lower grade tumor and high grade tumor to get an insight into the biology of astrocytoma development and progression. We have further identified a set of GSC (glioma stem-like cell) specific RBPs, from which an RBP prognostic signature was derived.

## RESULTS

### Integrated genome analysis reveals genetic and epigenetic alterations in RBPs in glioblastoma

**Figure 1:**
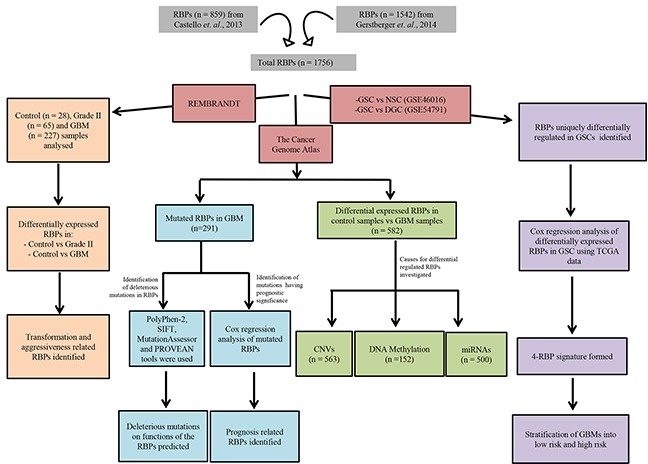
Flow chart showing the strategy employed to perform various analyses in this study 1756 RBPs used in this study were catalogued from two papers, Castello *et. al*., 2012 and Gerstberger *et. al*., 2014 [8, 9]. The three main branches indicate the three main foci of the study, including identification of transformation and aggressiveness related RBPs, genetic and transcriptional changes observed for RBPs in GBM including their causes, and GSC specific RBPs. The numbers in brackets indicate the samples examined to perform the specific analysis. GSC: glioma-like stem cells, NSC: normal neural stem cells, DGC: Differentiated glioma stem cells.

To elucidate the various aspects of RBP biology in GBM, we derived a comprehensive list of 1756 RBPs (Supplementary Table 1) from Castello *et al*., 2012 and Gerstberger *et al*, 2014 [8, 9] for further investigation. These proteins are either having RNA binding domains or are identified in the interactome of RNA and proteins in human cells. We carried out an integrated bioinformatics analysis to identify the RBPs that are altered in glioma due to genetic and epigenetic mechanisms. The strategy employed is depicted in Figure 1. Using these approaches we identified deleterious mutations and their association with prognosis, RBPs that are relevant to glia transformation and progression and that are uniquely expressed in glioma stem-like cells (GSC). We also developed an RBP signature from four prognostic RBPs and found it to be an independent predictor of survival in glioblastoma.

### Genetic alterations of RNA binding proteins in GBM

**Figure 2:**
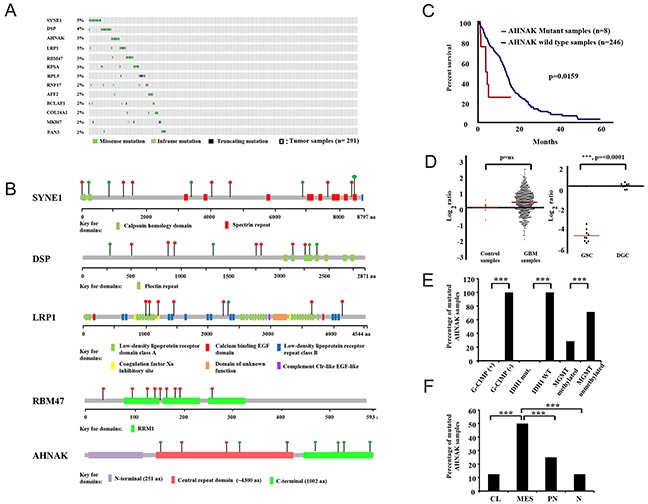
Genetic alterations in RBPs in GBM **(A)** Graphical representation of RBPs that are harboring non-synonymous alterations in more than 2% of the tumor samples. Each grey bar represents one GBM sample. Green bar represents the samples where a particular RBP has missense mutation, whereas black bar represents the sample where truncating mutations are identified in an RBP in GBM sample. **(B)** Graphical representation of coding region of top 5 mutated genes. The mutations are shown by green or red circles. Mutations shown in green circles represent the missense mutations (as per cBioPortal data), while those in red circles represent deleterious mutations as predicted by minimum of two tools from PolyPhen-2, SIFT, MutationAssessor and PROVEAN web server. Figures for SYNE1, DSP, LRP1 and RBM47 were obtained from cBioPortal and modified. Graphical representation of AHNAK was adapted from Davis *et. al*., 2014 [79]. The figures are not drawn to scale. **(C)** Kaplan Meier graph showing difference in survival between patients having wild type and mutant AHNAK protein. **(D)** Transcript levels of AHNAK in control and GBM samples (left); and in GSC and DGC using GSE54791 (right). **(E)** Bar diagram showing the percentage of mutated AHNAK samples with distinct characteristics, namely G-CIMP positivity or negativity, IDH1 wild type or mutant samples and MGMT methylated or unmethylated samples. **(F)** Bar diagram showing the percentage of samples with AHNAK mutations in different subtypes of GBM. CL : Classical, MES: Mesenchymal, PN : Proneural, N Neural.

To identify genetic alterations, we investigated the occurrence of mutations in RBPs using whole exome sequencing (WES) data of GBM samples derived from TCGA. We hypothesized that deleterious mutations in RNA binding domains, regulatory domains or other domains needed for protein-protein interaction may render oncogenic or tumor suppressive functions for the RBPs. We searched for RBPs harbouring genetic alterations including mutations and InDels (insertions or deletions) in their coding region. Analysis of TCGA WES data of 291 GBM samples revealed that there were 651 RBPs harbouring non-synonymous alterations in at least one GBM sample, while 13 of them were altered in more than 2% of samples (Figure 2A; Supplementary Table 2A and 2A). Additional investigation of WES data generated from our laboratory of six established glioma cell lines [10], we identified 53 RBPs that are mutated at least in one of the cell lines (Supplementary Table 2C). Among the 13 most mutated RBPs in GBM, 5 of them were also found to be mutated in these cell lines (Supplementary Figure 1). One of these five RBPs, AHNAK also harboured an insertion in one of the cell lines (Supplementary Figure 1).

Unlike genes like TP53, EGFR and PTEN which carry mutations in high proportion of GBM tumor samples (34%, 33% and 39% respectively) [11], RBPs are mutated in lesser proportion of tumor samples. The top most mutated RBP SYNE1 carried the mutation in 4.8% of the tumor samples analysed. The occurrence of mutations in SYNE1 in GBM has also effects on gene expression of other genes [12]. PolyPhen-2, SIFT, MutationAssessor and PROVEAN web server, which predict effects of mutations on the protein functions were used [13–16] to identify the potentially deleterious mutation in these mutated RBPs (Supplementary Table 2D). Out of top 13 RBPs, which had mutations, 11 of them were predicted to have potential deleterious mutation. The top five mutated RBPs and the predicted deleterious mutations (predicted minimum by two tools) are shown (Figure 2B). Moreover, we checked for the reported role of wild type and mutated proteins in literature (Supplementary Table 2E). Further, survival correlation between wild type and mutant patient samples for these 13 RBPs identified AHNAK alone as a survival predictor with mutants having poor prognosis (Supplementary Table 2F). Patients with mutations in AHNAK had a poor prognosis compared to those with wild type AHNAK gene (median survival of patients with AHNAK mutation = 4.16 months, median survival of patients with wild type AHNAK = 13.53 months) (Figure 2C). Although the AHNAK transcript levels was found to be similar in GBM compared to control brain samples (Figure 2D), we found a significantly lower level in GSCs when compared to the differentiated glioma cells (DGCs) (Figure 2D). It is interesting to note that all of the G-CIMP-negative and IDH1 wild type patients harboured AHNAK mutations. Moreover, majority of the AHNAK mutated samples also exhibited unmethylated MGMT promoter status (Figure 2E). We also found a higher occurrence of mesenchymal type of GBM in AHNAK mutant samples (Figure 2F). Further, both two-factor multivariate and multifactor multivariate with age, G-CIMP, MGMT or (and) IDH1, identified AHNAK mutation as an independent poor prognosticator (Table 1). Collectively, we were able to identify the RBPs that were carrying non-synonymous mutations in their coding region, and further categorize the subset of these mutations that may render the proteins non-functional.

**Table 1:** Multivariate cox regression analysis for AHNAK mutated samples and other prognostic markers using TCGA cohort

Factor	No. of patients	HR	B (coefficient)	P value
**I. Univariate analysis TCGA dataset**				
Age	254	1.037	0.036	<0.0001
G-CIMP	253	0.220	-1.512	0.003
MGMT	192	0.552	-0.594	0.002
IDH	254	0.240	-1.426	0.005
AHNAK	254	2.646	0.973	0.020
**II. Multivariate analysis with TCGA dataset**				
Age	254	1.037	0.037	<0.0001
AHNAK		2.667	0.981	0.020
G-CIMP	253	0.224	-1.496	0.003
AHNAK		2.467	0.903	0.032
MGMT	192	0.550	-0.598	0.002
AHNAK		2.605	0.957	0.039
IDH	254	0.244	-1.409	0.005
AHNAK		2.502	0.917	0.029
**III. Multivariate analysis of all the markers in TCGA dataset**				
Age	192	1.041	0.041	<0.0001
G-CIMP		0.000	-10.182	0.954
MGMT		0.713	-0.339	0.081
IDH		10004.361	9.211	0.959
AHNAK		2.562	0.941	0.043

### Transcriptional regulation of RBPs in GBM

**Figure 3:**
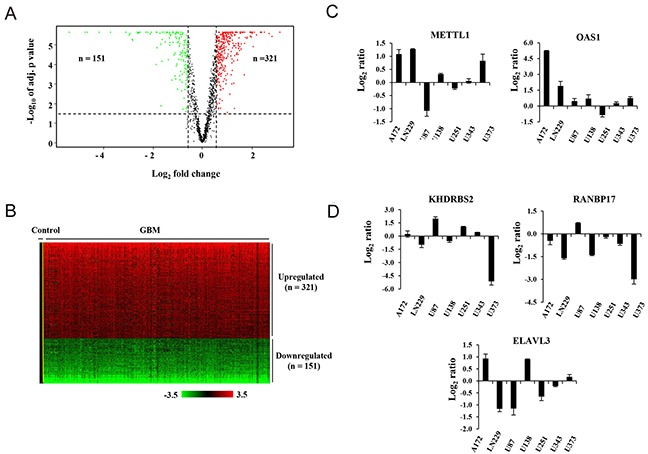
Transcriptional aberrations observed in RBPs in GBM **(A)** Volcano plot representing upregulated (red dots), downregulated (green dots) and unregulated (black dots) RBPs in GBM samples (n = 572) as compared to control samples (n = 10) using TCGA data. The horizontal dotted line demarcates the genes having significant expression difference (p-value < 0.05). Vertical dotted lines represent the applied cut off (absolute fold 1.5) for identifying differentially regulated genes. **(B)** Heat map of the 472 differentially expressed RBPs in GBM samples when compared to control brain samples. A *dual-color code* was used, with *red* and *green* indicating upregulated and downregulated RBPs, respectively. 321 RBPs were found to be upregulated and 151 RBPs were found to downregulated in GBM compared to control samples. The *yellow line* separates control samples from GBM samples. **(C, D)** Transcript levels (in Log_2_ ratio) of selected upregulated (C) and downregulated genes (D) in the mentioned glioma cell lines relative to Immortalized Human Astrocytes (IHA).

Out of the 1756 RBPs catalogued, the transcriptome information was available only for 1462 RBPs in TCGA. (Agilent platform) We found that 472 RBPs of this subset was differentially regulated between the control and GBM samples (Figure 3A); of which 321 were upregulated and 151 were found to be downregulated (Figure 3B, Supplementary Table 3). We thus observed a significant proportion (68%) of the differentially regulated RBPs to be upregulated in GBM samples *versus* control tissues (p < 0.0001). This was interesting because the analysis of the whole transcriptome revealed approximately equal proportion of upregulated (n = 3500) and downregulated (n = 3704) genes in GBM compared to control brain samples (p = 0.688) (Supplementary Figure 2A). The differential transcriptome analysis of RBPs is also validated in REMBRANDT, GSE22866 and GSE7696 data sets. We found 95-97 % of 472 RBPs to be similarly differentially regulated in these data sets (Supplementary Figure 2B, 2C, 2D and Supplementary Table 3). Hence, using multiple datasets we were able to conclude that major proportion of RBPs is upregulated in GBM when compared to control tissue samples. We also validated the expression pattern of two upregulated (METTL1 and OAS1) (Figure 3C) and three downregulated genes (KHDRBS2, RANBP17 and ELAVL3) in glioma cell lines using qRT-PCR (Figure 3D). While we found variation in their expression pattern in different glioma cell lines, there was in principle a similar expression pattern in some cell lines (Figure 3C and 3D).

**Figure 4:**
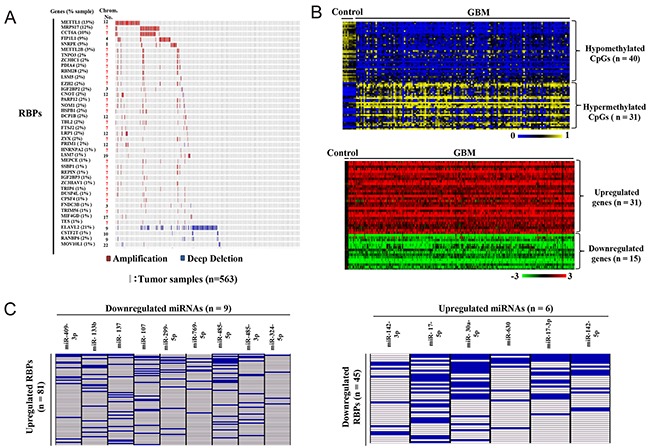
Probable causes for aberrant expression of RBPs in GBM **(A)** Graphical representation of RBPs with copy number variation in GBM samples compared to control samples. The samples in red and blue indicate the RBPs that are amplified and deleted respectively. The numbers in brackets indicate the percentage of samples in which a particular RBP had CNVs. The chromosomal location of a particular CNV is also indicated. **(B)** Heat maps representing the selected differentially expressed RBPs which are also differentially methylated. A dual-color code was used for methylation related heat map (top), wherein blue and yellow indicate hypomethylated CpGs and hypermethylated CpGs respectively corresponding to the upregulated and downregulated genes shown in dual color (red-green) expression related heat map (bottom). A dual-color code was used for expression related heat map, wherein red and green indicate upregulated and downregulated RBPs respectively. Their expression pattern in GBM versus control samples is shown in the heat map in the bottom panel, while their corresponding differentially methylated CpGs are represented in the heat map in the top panel. **(C)** Tabular representation of RBPs and the putative targeting miRNAs. Differentially regulated miRNAs predicted to target both upregulated and downregulated RBPs were identified using miRwalk. Only those miRNAs which were predicted to target the input differentially regulated RBP in seven or more than seven algorithms in miRwalk and having reciprocal regulation as compared to targeted RBPs are represented here. The blue boxes indicate the predicted miRNA-RBP targeting pair, while white boxes correspond to non-targeting miRNA-RBP pairs. Left: Upregulated RBPs predicted to be targeted by downregulated miRNAs; right: downregulated RBPs predicted to be targeted by upregulated miRNAs.

We next investigated the possible mechanisms behind differential regulation of RBPs. Analysis of copy number variation data from TCGA revealed that out of the 321 upregulated RBPs, 37 were amplified, while from 151 downregulated RBPs, 5 were deleted in more than 1% of tumors (Figure 4A, Supplementary Table 4A). At a 10% cut off, three genes METTL1, MRPS17 and CCT6A were found to be amplified while ELAVL2 was found to be deleted (Figure 4A). Interestingly, the segment containing METTL1 (12q14) has previously been reported to be amplified in GBM [17]. Further, we found that most of the amplified RBPs were present on chromosome 7, which is known to carry amplification of many genes (especially EGFR and MET) in GBM (Supplementary Table 4A) [18]. From our analysis we conclude that 11.5% of upregulated RBPs were found to be amplified, while 3% of downregulated RBPs were deleted at their chromosomal location.

Next, we investigated the epigenetic mechanisms behind differential regulation of RBP transcripts. Differential methylation analysis using TCGA 450K array, revealed that out of the 472 differentially regulated RBPs, 45 RBPs had differential methylation in GBM samples as compared to control brain samples. We found that there were 30 genes, corresponding to 40 CpGs which were hypomethylated, while 15 genes, corresponding to 31 CpGs, were hypermethylated (Figure 4B, Supplementary Table 4B). These results were further strengthened by validation in our patient cohort (GSE79122) and GSE60274. We found all the genes to be similarly methylated in GSE79122 data set, while approximately 85% genes were similarly methylated in GSE60274 (Supplementary Figure 3A and 3B, Supplementary Table 4B). We then validated the methylation status of three selected hypermethylated genes by using methylation inhibitor 5-aza-2-deoxycytidine (DAC). We found that KHDRBS2 and RANBP17, which are downregulated in LN229 and U373 cell lines (Figure 3D) were re-expressed to varying levels upon DAC treatment in both cell lines (Supplementary Figure 3C and 3D). On the contrary, DAC treatment induced re-expression of ELAVL3 in LN229 cells wherein the expression of this is downregulated (Supplementary Figure 3D and Figure 3D). However, the re-expression of ELAVL3 was not observed in U373 cells wherein this gene was not downregulated (Supplementary Figure 3C and Figure 3D). Collectively, these results conclude that downregulation of KHDRBS2, RANBP17 and ELAVL3 in glioma is indeed due to DNA methylation.

To study the role of miRNAs in regulation of RBP transcript levels, we used miRwalk [19] to identify miRNA that can target differentially regulated RBPs. As miRNAs are also shown to regulate the transcript levels of their target genes, we focused on the miRNAs that are downregulated in GBM, but predicted to target upregulated RBPs and *vice versa*. Using TCGA expression database for mRNA and miRNA, we found that 9 downregulated miRNAs can putatively target 81 upregulated RBPs in GBM (Figure 4C-left panel; Supplementary Table 4C). Conversely, 6 upregulated miRNAs can putatively target 45 downregulated RBPs (Figure 4C-right panel; Supplementary Table 4C). This reflects a large proportion of differentially regulated RBPs that may be regulated by miRNAs during GBM development. We also report 28 validated miRNA-RBPs pairs wherein the mentioned miRNAs have been shown to target the stated RBPs in literature (Supplementary Table 4C). Altogether, in our analysis we found that of all the three factors analysed, miRNAs play a major role in regulation of differentially expressed RBPs (Supplementary Figure 4A and 4B).

### RBPs as a cause of astrocyte transformation and glioma progression

**Figure 5:**
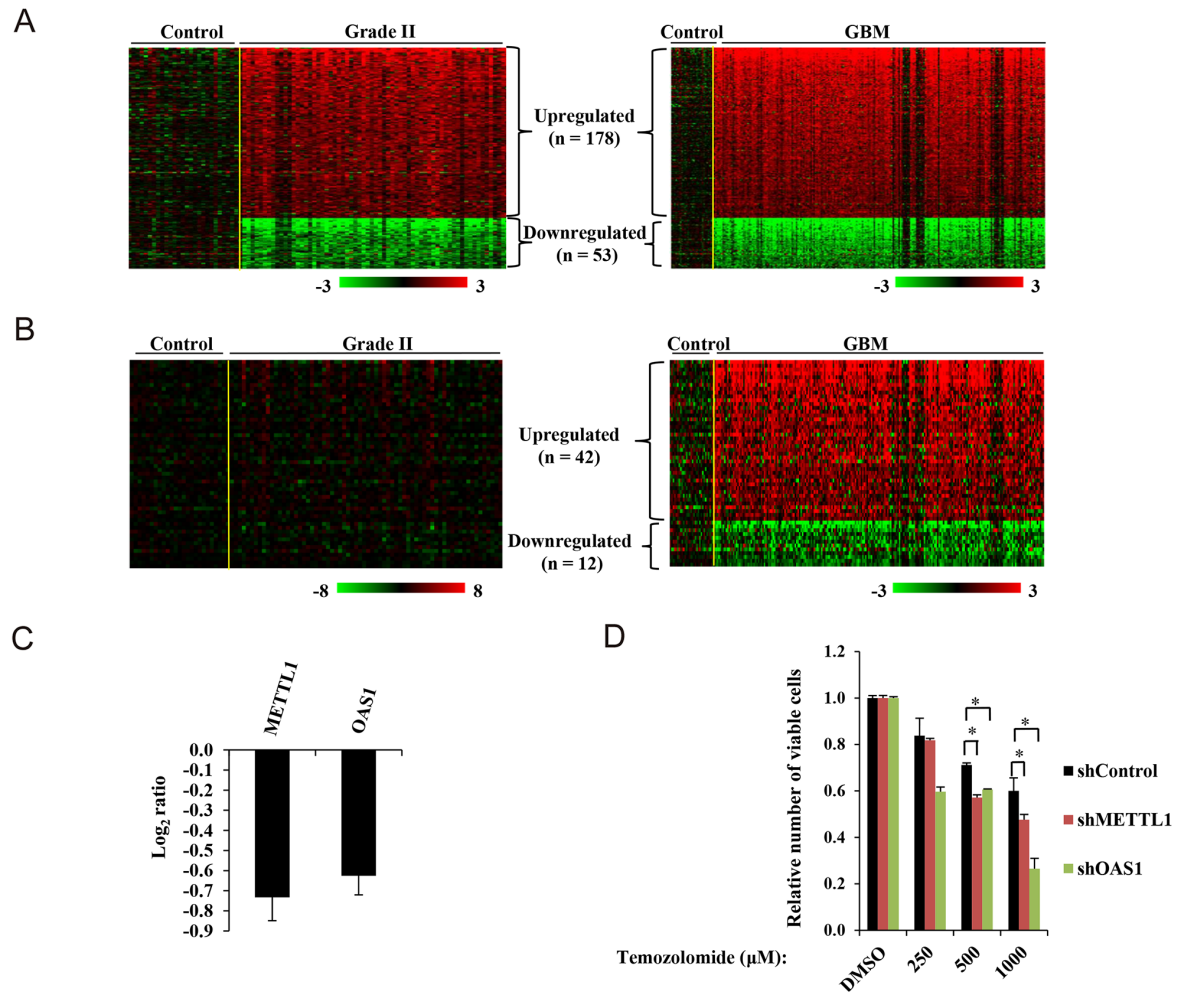
Transformation and aggressiveness related RBPs in GBM **(A)** Heat maps representing the differentially regulated genes in Grade II *versus* control samples (left panel) which retain similar pattern of expression in GBM (right panel). **(B)** Heat maps depicting the genes which are uniquely differentially regulated in GBM samples *versus* control samples (right panel), but not showing any significant difference in Grade II and control samples (left panel). A dual color-code was used with red and green indicating upregulated and downregulated RBPs respectively. A *yellow line* separates control samples from grade II or GBM samples in the given heat maps. **(C)** Transcript levels of METTL1 and OAS1 were quantified using qRT-PCR in LN299 cells where shRNA mediated knockdown of METTL1 and OAS1 has been performed respectively and represented. **(D)** Bar graph representing relative number of viable cells in control and indicated knockdown LN229 cells at the mentioned Temozolomide concentrations. The number of viable cells in shControl, shMETTL1 and shOAS1 conditions in the presence of temozolomide was plotted relative to the number of viable cells in the respective DMSO treated condition.

The formation of secondary GBM involves malignant progression from grade II and III astrocytoma [20]. Retrospective analysis of molecules in different stages of tumors in its development can give important insights into the key players of immortalization, transformation and aggressiveness. We hypothesized that comparison of RBP transcripts in control brain samples, grade II and GBM tumors may give a glimpse of RBPs that may be required for events leading to initial astrocyte transformation. We assumed that these will be differentially regulated in control samples and grade II glioma and their expression pattern will be retained in GBM samples as in grade II tumors. Further, the RBPs which may be responsible for aggressive behavior (malignant progression) of GBMs may be differentially regulated uniquely in GBM, but not in grade II. We only incorporated grade II and GBM samples in our analysis while grade III samples were not considered because of its mixed molecular nature. This study revealed that 231 RBPs are differentially regulated between control brain and grade II samples and their expression pattern is retained in GBM samples. These may be implicated in early events of transformation of normal astrocyte (Figure 5A, Supplementary Table 5A). This analysis also revealed that 176 RBPs were differentially regulated specifically in GBM and were not altered in grade II compared to control brain samples (Figure 5B, Supplementary Table 5B). These RBPs may be implicated in phenotypes like migration, invasion, angiogenesis and chemoresistance which contribute to aggressiveness of GBM. Taken together, we identified RBPs which may be playing a role in initial events like transformation of normal cells and are essential even in the later stages of the tumor. We also identified another class of RBPs which may be required only during the aggressive stages of tumor, but their expression may not be required at the initial stages of tumor formation.

Further, to experimentally validate the contribution of RBPs in aggressiveness of GBM tumors, we selected *Methyltransferase-like 1* (METLL1) and *2′-5′-Oligoadenylate Synthetase 1* (OAS1) which identified as aggressiveness related RBPs and were specifically upregulated in GBM tumors as compared to control samples. We performed lentiviral shRNA mediated knockdown of METTL1 and OAS1 in LN229 glioma cells. Knockdown of these genes in LN229 glioma cells was confirmed by the reduced transcript levels using qRT-PCR (Figure 5C). In chemosensitivity assays performed using MTT; we found that knockdown of these genes imparted chemosensitivity to Temozolomide in LN229 cells (Figure 5D).

### Role of RBPs in glioma stem-like cell (GSC) maintenance and GBM prognosis

**Figure 6:**
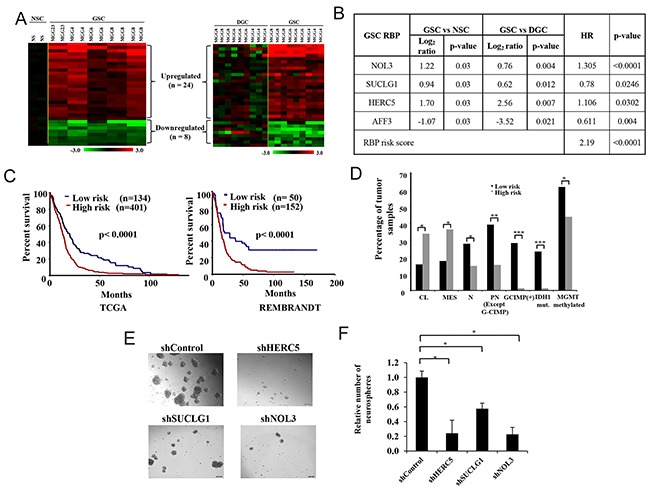
GSC specific RBPs compared to NSCs and DGCs **(A)** Heat map representing differentially regulated RBPs in NSC *versus* GSC (left) and DGC *versus* GSC (right). Datasets used were GSE46016 (NSC *versus* GSC) and GSE54791 (GSC *versus* DGC). Out of 1756 RBPs, the expression of 1621 and 1614 were available in GSE46016 and GSE54791 respectively. The experiment was performed in replicates and the expression value of each replicate is represented. A dual-color code was used, with red and green indicating upregulated and downregulated RBPs, respectively **(B)** Table showing genes which are having significant univariate values in GBM samples, along with their hazard ratios and p-value. **(C)** Kaplan Meier curve showing stratification of GBM samples into low-risk and high-risk patients using the four RBPsignature. **(D)** Bar graph showing the percentage of GBM tumor samples containing IDH1 mutant (mut), G-CIMP (+):G-CIMP-positive, methylated MGMT (promoter) and different subtypes of GBM tumor samples features in high-risk and low-risk groups (as stratified in c). CL : Classical, MES: Mesenchymal, PN : Proneural, N Neural. **(E)** Representative micrographs of neurospheres formed upon knockdown of mentioned genes. **(F)** Quantification of the number of spheres formed in the indicated conditions. The number of spheres in shHERC5, shSUCLG1 and shNOL3 are plotted relative to shControl condition.

GSCs are related to many of the aggressive properties of cancers like migration, invasion, angiogenesis and chemoresistance [21–25]. Thus, an important area of research is on the genes that are essential for GSC survival and maintenance. In order to delineate the expression of RBPs specifically in GSC, we investigated two data sets GSE46016 and GSE54791, which carried the transcriptome profile of neuronal stem cells (NSC), three GSCs and their differentiated glioma cells (DGCs). We were specifically interested in RBPs that are differentially regulated in GSCs over DGCs and NSC. Analysis revealed that 24 RBPs are upregulated and 8 RBPs are downregulated specifically in GSCs when compared to NSCs and DGCs (Figure 6A, Supplementary Table 6). These RBPs may be implicated in pathways indispensible for GSC maintenance.

As the GSCs contribute to the aggressiveness of the tumor, and are implicated in its resistance and relapse, it is reasonable to think that a signature of genes uniquely regulated in GSCs may be able to predict patient survival in GBM. Univariate cox regression analysis of GSC specific RBPs (n = 32) using TCGA expression data set (Agilent platform) revealed that four genes- HERC5, NOL3, SUCLG1 and AFF3 predicted survival (Figure 6B; Supplementary Table 6). An RBP risk score calculated for each patient by combining the effect of each of these 4 RBPs using a risk score formula (mentioned in Materials and Methods section) was found to be a poor prognostic indicator (Figure 6B). RBP risk score was also able to divide the GBM patients into two groups, high-risk (median survival of 12.87 months) and low-risk (median survival of 19.77 months), with a median survival difference of 6.9 months in TCGA dataset (p= <0.0001, HR= 1.648, B= 0.499) (Figure 6C). RBP risk score generated from REMBRANDT dataset also divided GBM patients into low-risk and high-risk with significant difference in their survival (Figure 6C).

Multivariate cox regression analysis using TCGA expression data (Agilent platform) was carried out to test the strength of the RBP risk score in its ability to predict survival (Table 2). A two way multivariate analysis involving RBP risk score against age, G-CIMP, MGMT promoter methylation, IDH1 mutation identified RBP risk score as an independent predictor of patient survival (Table 2). Additionally, in a multivariate analysis involving all five markers against 299 GBM patients (who received any type of chemotherapy), RBP risk score was able to independently predict patient survival with nearing significance (Table 2). However, a similar analysis of 209 GBM patients, who only received Temozolomide as chemotherapy revealed that RBP risk score is indeed an independent predictor of GBM patient's survival (Table 2). Collectively, we made a 4-gene RBP signature, which is not only an independent predictor of survival in GBM but was also able to stratify the GBM patients into low- and high-risk groups with significant difference in survival.

**Table 2:** Multivariate cox regression analysis for RBP signature and other prognostic markers using TCGA cohort

Factor	No. of patients	HR	B(coefficient)	P value
**I. Univariate analysis TCGA dataset**				
Age	535	1.04	0.04	<0.0001
G-CIMP	525	0.32	-1.15	<0.0001
MGMT	344	0.67	-0.40	0.0021
IDH	416	0.37	-0.99	0.0001
RBP risk score	535	2.19	0.78	<0.0001
**II. Multivariate analysis with TCGA dataset**				
Age	535	1.03	0.03	<0.0001
RBP risk score		1.61	0.48	0.0032
G-CIMP	525	0.39	-0.95	<0.0001
RBP risk score		1.45	0.37	0.0421
MGMT	344	0.73	-0.32	0.0162
RBP risk score		2.69	0.99	<0.0001
IDH	416	0.50	-0.69	0.0109
RBP risk score		1.87	0.63	0.0014
**III. Multivariate analysis of all the markers in TCGA dataset**				
Age	299	1.04	0.04	<0.0001
G-CIMP		0.35	-1.04	0.3134
MGMT		0.80	-0.23	0.1109
IDH		2.19	0.78	0.4516
RBP risk score		1.64	0.49	0.0552
**IV. Multivariate analysis of all the markers in TCGA dataset (patients treated with Temozolomide)**				
Age	209	1.03	0.03	<0.0001
G-CIMP		0.0004	-7.78	0.922
MGMT		0.653	-0.427	0.017
IDH		2541.01	7.840	0.922
RBP risk score		2.54	0.934	0.007

We carried out additional investigations to gain further insights into biological meaning of patient survival prediction by RBP risk score. We compared some of known predictors of GBM survival with our RBP risk score using TCGA GBM data. While the high-risk group as defined by RBP risk score is enriched for classical and mesenchymal gene expression subtype, the low-risk group is enriched with neural and proneural gene expression subtype of GBM (Figure 6D). Further we also found that the low-risk group is enriched with GBM patients that belong to G-CIMP-positive group, harboured IDH1 mutation and had methylated MGMT promoter (Figure 6D). Additionally, we also checked the importance of the three upregulated RBP signature genes (HERC5, SUCLG1 and NOL3) in GSC maintenance. We performed shRNA mediated knockdown of these genes in MGG4 patient derived GSCs. We observed that knockdown of each of the three genes significantly impaired the neurosphere formation (Figure 6E and 6F).

**Figure 7:**
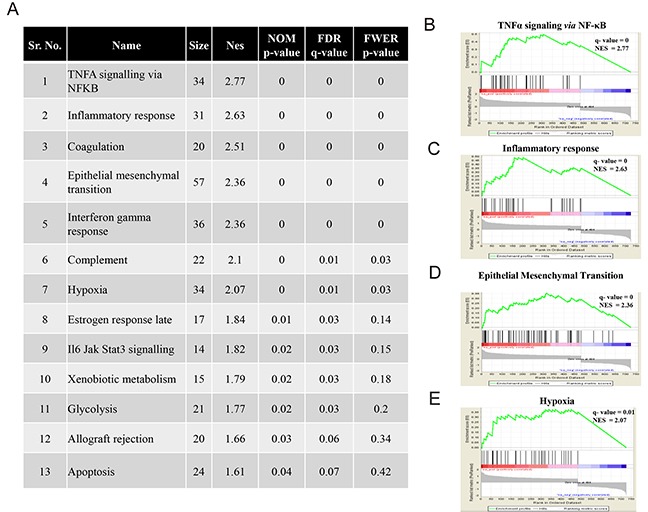
Significant pathways enriched in high-risk patients **(A)** Significant pathways enriched in high-risk patients using GSEA of differentially regulated genes in high-risk and low-risk patients. **(B, C, D, E)** GSEA representation of selected significant enriched pathways in high risk group, namely TNFa signalling via NFκB **(B)**, inflammatory response **(C)**, Epithelial mesenchymal transition **(D)** and hypoxia **(E)**.

Next, we carried out Gene set enrichment analysis (GSEA) using the differentially regulated genes (using TCGA expression data-Agilent platform) between the low-risk and high-risk GBM patients (Supplementary Table 7) on an available dataset from MSigDB (mentioned in Materials and Methods). There were 13 pathways that were positively enriched in high-risk group (Figure 7A). At a higher cut-off using FWER p-value, we got seven pathways enriched in high-risk group and these pathways included NFκB, inflammatory response, epithelial-mesenchymal transition, and hypoxia (Figure 7B–7E). Thus, our analysis revealed RBPs that may be important for GSCs, and also as indicators of survival in GBM patients. Moreover, we developed a 4 RBP prognostic signature which effectively stratifies GBM patients into high-risk and low-risk groups.

## DISCUSSION

The three major steps at which the gene expression can be modulated would include transcription, RNA processing and post-translational modification of proteins. At RNA processing levels, some of the steps like 5′ capping, addition of poly-A at the 3′ end, splicing and subsequent translation have been extensively studied [26–31]. With the advent of high throughput techniques like mass spectrometry and sequencing, about 1756 RBPs have been identified [8, 9]. While few reports have been published underscoring the importance of RBPs as global regulators and their role in cancers [3, 7, 32–34], the function of vast majority of these RBPs is yet to be explored. This study gives a comprehensive landscape of RBP regulation and their importance in glioma development and progression.

Our study emphasizes that the RBPs are deregulated in GBM. With the prior knowledge that genetic alterations (including mutations) in RBP may lead to various diseases like cancer, we analysed for the RBPs that are mutated in GBM in their coding region. Our study also identifies mutations in RNA binding domains of RBPs which may prove to be deleterious for their functions. In our study, we found mutations in the RNA binding domains of RBM47 and RPL5, which are suggested to be tumor suppressors in a few cancers [35, 36]. Thus, the loss of the function of these proteins may be imperative for tumor progression [36]. Another interesting mutated protein found in our analysis was AHNAK. AHNAK is a large protein of 700 kDa in size and it binds to R-smad in response to TGF-β mediated down regulation of c-Myc expression and cell growth retardation [37]. AHNAK has also been shown to inhibit induced pluripotent stem cells (iPSC) formation [38]. Moreover, it was reported by Sheppard *et.al*, that mutations in AHNAK were common in metastatic melanoma patients and also correlated with poor outcome [39]. We found that GBM patients having mutated AHNAK survived lesser. The expression of AHNAK was found to be lesser in GSC compared to DGC suggestive of a cancer stem cell inhibitory function in glioma. It was also interesting to note that AHNAK mutated GBMs, who have poor survival, were enriched in poor prognostic groups as identified by G-CIMP, IDH1, MGMT promoter methylation and also in mesenchymal subtype of GBM.

In our analysis, we found that major proportion of RBPs were upregulated in GBM samples. This agrees with the previously published literature, which shows that majority of the RBPs are upregulated in various cancers as compared to their normal counterparts [40]. This suggests that majority of them may have a pro-tumorigenic role. Interestingly, we found members of RBPs that belong to distinct gene families to be differentially regulated. Some notable families included nucleic acid editing enzymes (ADAR and APOBEC members), ELAVL family members and IGF2BP family members. ADARB1 and ADARB2 enzymes edit adenosines to inosines on their RNA substrates. These enzymes had significantly lower expression in GBM when compared to control samples in our analysis. Indeed, literature suggests that they possess tumor suppressor activity [41–43]. On the other hand, DNA editing enzymes like APOBEC3C, APOBEC3F, APOBEC3G and APOBEC3H were upregulated in GBM in this study. APOBEC3 have been implicated in enhancing survival of cancer cells, by efficiently repairing DSB repair, thus preventing cell death, and also by contributing to accumulation of mutations that may drive tumor progression [44]. Another fascinating observation was made regarding the expression status of ELAVL family members in GBM. Our study found that the expression of ELAVL1 was significantly high in tumor samples, while that of other three members (ELAVL2, ELAVL3 and ELAVL4) was significantly low in tumors. This confounding result motivated us to check the potential role of these proteins in cancers. Though ELAVL1 (HuR) is a well-studied protein, limited reports exist for roles of the other three family members in cancers [45–48]. The downregulated members are established to have neuronal restricted expression and some of them have been reported to have a putative tumor suppressor role. Mansfield *et. al*., in 2012, had demonstrated that during neuronal differentiation, ELAVL2, ELAVL3 and ELAVL4 cause alternative polyadenylation of HuR and hence suppress its translation, leading to a non-proliferative state [49]. In GBM scenario, we hypothesize that downregulation of ELAVL2, ELAVL3 and ELAVL4 may be preferred to further enhance the translation of HuR, leading to a less differentiated and highly proliferative state of cancer cells.

From our analysis, it was evident that RBPs have aberrant expression in GBM patients. Causal mechanisms include genetic, epigenetic, post transcriptional and post translational regulations. We investigated into few of the causes which might lead to alteration in expression pattern of RBPs in GBM, including copy number variation, differential methylation and miRNA mediated regulation. Our analysis gives a concise list of these factors that may be responsible for differential expression of these RBPs either alone, or in combination. The results of our analysis also included some of the known RBPs regulated by these factors. In case of copy number variation, we found METTL1 to be amplified and over-expressed in other cancers including lung cancer [50]. The amplification of the segment in which the gene resides harbours other genes like CYP27B1, FAM119B, TSFM and AVIL which are co-amplified in several tumors due to amplification of this region. This protein is known to methylate tRNAs. Moreover, its expression may impart chemo-resistance in HeLa cells [51]. ELAVL2 is the gene found to be deleted in maximum percentage of cases in our analysis. ELAVL2 is located at the chromosome band 9p21.3, where CDKN2A (a well-known tumor suppressor) is also located. Further, it is shown to act as a tumor suppressor in GBMs and glioma initiating cells [48].

One of the epigenetic factors that we studied here was methylation of RBPs. Methylation changes in the gene may have severe effects on the gene expression. As an established fact, in most cases, hypermethylation of promoters of the genes leads to repression of transcription and *vice versa*. In our study, we found a significant contribution of methylation in regulation of expression of the RBPs examined in GBM. KHDRBS2 was found to be the most downregulated and hypermethylated RBP. Hypermethylation of CpG sites in this gene was reported as a hallmark of CIMP phenotype in renal cell carcinoma. It was one of the genes conferring the CIMP phenotype in GBM too. On the other hand, IGF2BP3 was found to be the most hypomethylated and upregulated gene in TCGA and also in our cohort. This gene has been used as a biomarker for advanced malignancies including GBM. It is also shown to be an oncogene in many cancers, contributing to various hallmarks of cancer [34, 52–55].

In our analysis, we found miRNAs to have maximum impact on the aberrant RBP expression in GBMs. We revealed that 15 miRNAs may be responsible for the differential expression of 126 RBPs. Some of the miRNA-RBP pairs found in our analysis were already reported in the literature. It was demonstrated using luciferase assays that miR133-b represses CPNE3 in prostate cancer [56]. In downregulated miRNA and upregulated RBP pairs, miR142-5p was reported to target ATXN [57], while miR-142-3p targets BCLAF1 and PUM1 [58]. These reports further strengthen the reliability of our findings. We were intrigued to notice two IGF2BPs, IGF2BP3 and IGF2BP2 that were found to be upregulated in GBM, and may be regulated by all the three factors, namely copy number alterations, DNA methylation and miRNA mediated regulation. This may underscore the importance of upregulation of these RBPs in GBM, such that regulation by one or multiple mechanisms was imposed to ensure that the expression of these RBPs was high in GBM tumor samples.

RBPs are implicated in global level changes, and may hence play a role in initial transformation events or later malignant progression to advanced stages. Initial multiple genetic changes required to convert a normal cell to a transformed cell, is followed by a process of Darwinian selection where cells carrying beneficial genetic or epigenetic changes are selected progressively, as the tumor advances. The events that aid in immortalization, transformation and proliferation may be the ones that are differentially regulated at the early stages of the tumor and are retained at the later stages too. On the contrary, some of the genes are required for later stages of tumor. These will be the genes that are only expressed in aggressive stages of tumor. We identified 231 RBPs that may be implicated in initial transformation events, while 176 RBPs that are differentially expressed only in high grade tumors, which may be important for their aggressive phenotype. Certainly, in our analysis, we found RBPs that were already reported to contribute to the aggressiveness of the high grade cancers. These included IGF2BP3 [34, 54], S100A4 [59, 60], METTL1 [51], NPM1 [61, 62], BST2 [63], and EIF4E2 [64]. We also validated the role of two upregulated and aggressiveness related RBPs (METTL1 and OAS1) in chemoresistance of LN229 glioma cells to temozolomide.

GSCs comprise a small proportion of the tumor cells, but are known to contribute to the aggressiveness of the tumors. We investigated and identified RBPs that are differentially and selectively present in GSCs rather than in normal neural stem cells and differentiated tumor cells. Further, we identified four RBPs that had prognostic significance in GBM tumor patients. Moreover, further analysis revealed that these four together had a better prognostic value as compared to any of them individually. This signature comprising of HERC5, NOL3, SUCLG1 and AFF3 was able to stratify patients into low and high-risk GBM patients. In depth understanding of these two groups at molecular level showed that number of G-CIMP-positive and IDH1 mutant patients were more in low-risk group. This corroborated with the earlier findings that patients with IDH1 mutation had better survival [65]. As IDH1 mutation alone is sufficient to create G-CIMP related methylome [66], it is easy to comprehend that G-CIMP-positive patients will be surviving better as also seen in our analysis. We also observed a significant reduction in neurosphere forming ability by patient derived GSCs when each of these genes (HERC5, NOL3 and SUCLG1) was silenced individually in these cells. NOL3 has been previously shown to increase apoptosis in the presence of imatinib treatment [67].

We performed pathway enrichment analysis to recognize pathways deregulated in identified high-risk patients. Hypoxia came as one of the deregulated pathways in high-risk patients. Hypoxia is known to contribute to GBM progression, therapy resistance and recurrence by providing a niche for maintenance of GSCs in tumors. Another process that was found to be active in high-risk tumors was EMT or Epithelial to mesenchymal transition. EMT is closely associated with migration and invasion of GBM cells, from the area of tumor formation. To our key interest was NFκB signalling pathway, which was enriched most significantly in the high-risk tumors. The pathway has already been established in the literature to regulate genes participating in the other aforementioned pathways and hence contribute to the aggressiveness of the disease [68, 69]. The key players included some crucial targets of this signalling pathway namely SOD2, VEGFA, IL6, IL8, TNC, CD44, CCL20 and ICAM1 [70]. Taken together, the enhanced activity of these pathways in high-risk patients gives an insight into the accuracy of the stratification of patients using our RBP signature and thus underscores its importance in the same.

To our knowledge, this study provides the first comprehensive view of aberrantly regulated RBPs in GBM and mechanisms which may lead to this aberrant regulation. It also gives insights into transformation and aggressiveness related RBPs, and those which may contribute to GSC related pathways. We believe that this meta-analysis encompassing various aspects of RBP biology in GBM may prove to be highly useful for future studies in this field. We also developed an RBP signature, which proved to be a reliable independent prognosticator in GBM. This could be helpful in clinics, while making decisions related to therapies that have to be administered to GBM patients.

## MATERIALS AND METHODS

### Cataloguing of RBP

A comprehensive list of 1756 RBPs used in this study was collated from data reported in Castello *et. al*., 2012 and Gerstberger *et. al*., 2014 [8, 9] (Supplementary Table 1). These RBPs were either reported to have known or predicted RNA binding domains or had been identified by their ability to bind to RNAs. This list is used for conducting all the analyses in this study.

### Mutational and InDels analysis for RBPs

TCGA level 2 somatic mutation data was downloaded and analysed (https://tcga-data.nci.nih.gov). Tools like PolyPhen-2, SIFT, MutationAssessor and PROVEAN web server, [13–16] were used to find the deleterious effects of the non-synonymous mutations found in RBPs in our analysis. cBioPortal (http://www.cbioportal.org/) [71] was used to graphically represent the RBPs genetically altered in more than 2% of the samples.

### Differential expression analysis

We obtained expression data for GBM samples at the TCGA web site (https://tcga-data.nci.nih.gov), REMBRANDT, GSE22866 [72] and GSE7696 [73, 74]. In case of TCGA, Level 3 data for Agilent platform (Agilent 244K Custom Gene Expression G4502A) was downloaded and subsequently used for analysis. For differential expression analysis, average of the values of all control samples was taken and this was subtracted from each of GBM sample value. Fold change of genes was calculated by subtracting average of control samples from average of GBM values. Statistical significance was calculated using Wilcox Mann-Whitney Test. Only the genes having more than or equal to 1.5 absolute fold change and significant p-value (p-value < 0.05; t-test: Mann-Whitney and additional Benjamini/Hochberg FDR correction was applied) were considered to be differentially expressed.

For GSC and DGC expression analysis for RBPs, GSE54791 [75] was used, while for GSC and NSC expression analysis, GSE46016 [76] was used. All GEO datasets were retrieved from NCBI GEO database. Expression data for 1614 RBPs was available in GSE54791 while that of 1621 RBPs was present in GSE46016. To calculate GSC specific differentially regulated RBPs, we first identified differentially regulated genes in GSC versus NSC (p-value <0.05; t-test: Mann-Whitney) and in GSC versus DGC (p-value <0.05; t-test: Mann-Whitney and additional Benjamini/Hochberg FDR correction was applied). The common upregulated and downregulated genes in both conditions in GSCs were then termed as GSC specific differentially regulated RBPs over the other two conditions. Only the genes having more than or equal to 1.5 absolute fold change and significant (p-value < 0.05) were considered to be differentially expressed.

### Methylation data analysis

Corresponding probes for differentially expressed genes (472 genes from TCGA analysis) were taken from TCGA-Illumina Infinium Human DNA Methylation 450K platform (https://tcga-data.nci.nih.gov). Data for control samples was used from GSE79122. Probes having differential beta values (a measure of methylation) were calculated by subtracting average beta value of each probe in control samples from average beta value of the same in GBM samples. Statistical significance was calculated using Wilcox Mann-Whitney Test. Only the probes having more than or equal to 0.3 absolute beta value difference and significant (p-value < 0.05) were considered to be differentially methylated. Similar analysis was performed on GSE79122 and GSE60274 [77] datasets. All GEO datasets were retrieved from NCBI GEO database.

### miRNA

Differentially expressed RBPs (from TCGA analysis) were taken as an input for miRwalk prediction [19]. A list of miRNAs targeting these RBPs in seven or more than seven algorithms was acquired. Further, using the expression data available for miRNA in TCGA (https://tcga-data.nci.nih.gov), we calculated the fold change of each miRNA as explained above for differentially regulated RBPs. Only the miRNAs that were reciprocally regulated with respect to their target RBPs were represented.

### Copy number variation

The data was obtained from cBioPortal (http://www.cbioportal.org/) and percentage of samples in which a particular RBP was amplified or deleted was calculated.

### Survival analysis

The uniquely differentially expressed RBPs in GSCs were used as an input for univariate analysis (using TCGA data). SPSS version 19.0 was used for univariate and multivariate analysis. Kaplan Meier survival analysis was performed using GraphPad Prism 5.0 version for Windows (GraphPad Software, San Diego, California USA, www.graphpad.com). The risk score for the signature was calculated using the following formula:

Risk score of a sample = ∑(cox regression coefficient of a particular RBP X log_2_ ratio value of expression of RBP).

### Gene set enrichment analysis (GSEA)

Differentially expressed genes in low-risk and high-risk patients as stratified by RBP signature were identified. This list was pre-ranked on the basis of fold change and used as an input to perform GSEA (GSEA 2.2.1) on “H: Hallmark gene set” encompassing 50 gene sets available in Molecular Signature Database (MSigDB). We acknowledge our use of the gene set enrichment analysis, GSEA software, and Molecular Signature Database (MSigDB) [78] (http://www.broad.mit.edu/gsea/).

### Cell lines, glioma stem-like cells and plasmids

Glioma cells (U87, U138, U251, U343, U373, LN229 and A172) and immortalized human astrocytes (IHA) were grown in DMEM supplemented with 10% FBS, Penicillin and Streptomycin. 293T, U87, U251 and U373 were procured from ECACC, while U138, U343, LN229 and IHA were procured from late Dr. Abhijit Guha. Priimary patient derived GSCs (MGG4) was a kind gift from Dr. Wakimoto H. (Massachusetts General Hospital, Boston). These cells were grown as neurospheres in Ultra-low attachment plates (Corning, U.S.A) in Neurobasal medium (Invitrogen, U.S.A.) supplemented with 3 mmol/L L-Glutamine (Invitrogen, U.S.A.), basic fibroblast growth factor (bFGF; 20 ng/ml, Promega), epidermal growth factor (EGF; 20 ng/ml; Promega), 1X B27 supplement (Invitrogen, U.S.A.), 0.5X N-2 (Invitrogen, U.S.A.), 2 μg/mL Heparin (Sigma, U.S.A.), penicillin, gentamicin and streptomycin in ultra-low attachment plates (Corning, U.S.A.). Neurospheres were passaged every 7 days using chemical dissociation kit (Catalog# 05707, STEMCELL technologies, U.S.A.). Fresh medium was added every 2-3 days.

shRNA plasmids against METTL1 (TRCN 0000035955-TRCN0000035958), OAS1 (TRCN 000000 5007-TRCN0000005011), HERC5 (TRCN 000000 4168- TRCN0000004171), SUCLG1(TRCN 0000048508- TRCN0000048512) and NOL3 (TRCN 0000118447-TRCN0000118451) were obtained as kind gift from Dr. Subba Rao and Dr. Saini from MISSION® shRNA Library (Sigma-Aldrich, U.S.A.).

### Lentivirus preparation

Pooled shRNAs for a specific gene (2μg) was co-transfected with psPAX and pMD2.G plasmids (3:1) in 50% confluent 293T cells using Lipofectamine 2000 (Invitrogen, U.S.A.). Media was changed after 6 h of transfection. Media containing viral particles was collected after 60 h of transfection. This supernatant was used to infect LN229 cells or MGG4 in presence of polybrene (Sigma-Aldrich, U.S.A.).

### 5-Aza-2′-deoxycytidine treatment

Glioma cell lines (U373 and LN229) were seeded at a density to reach 70% confluency at 24 hours. The cells were treated with 10, 20 and 50 μM of 5-aza-2′-deoxycytidine for 3 days and 5 days after 24 hours. The media was replaced with fresh media supplemented with 5-aza-2′-deoxycytidine every 24 hours. Total RNA was isolated at the indicated time points and the transcript levels of KHDRBS2, RANBP17 and ELAVL3 were assessed by qRT-PCR.

### RNA isolation and qRT-PCT

RNA was isolated using TRI-reagent (Sigma-Aldrich, U.S.A.) as per manufacturer's instructions. 2μg of RNA was converted into cDNA using High capacity cDNA reverse transcription kit (Life technologies, USA) according to the manufacturer's protocol. qRT-PCR was performed to quantitate the levels of transcripts in various conditions. ATP5G1 was used as an internal control and expression of genes were calculated using ΔΔ Ct method. Primers used in the study are *KHDRBS2 (forward)*: GCTTGGACCAAGAGGAAACTCC, *KHDRBS2 (reverse)*: CAAGTGGGCATATTTGGCTT CCC, *RANBP17 (forward)*: TGTTGATCGGGCTGGC AAGAGA, *RANBP17 (reverse)*: TGTTGGCTCTCCATA CCACCGT, *ELAVL3 (forward)*: TGCAGACAAAGCCAT CAACACCC, *ELAVL3 (reverse)*: GCTGACGTACAGGTT AGCATCC, *METTL1 (forward)*: TGGCTTCCAGAACAT CGCCTGT, METTL1 (reverse): TGTCCGCTTGAAATG TGGGTCG, *OAS1 (forward)*: AGGAAAGGTGCTTCCG AGGTAG, *OAS1 (reverse)*: GGACTGAGGAAGAC AACCAGGT.

### Chemosensitivity assays

Lentiviral shRNA infected LN229 cells were harvested and plated at 70-80% confluency in triplicates in a 96-well cluster plate. Temozolomide (Schering-Plough) at the indicated concentrations was added after 24 hours of plating. MTT (Sigma-Aldrich, U.S.A.) was used to assess the viability of cells under investigation after 72 hours of treatment. For MTT assays, 1.5 hours after MTT addition, the formazan crystals were dissolved in DMSO (200 μl) and measured as absorbance at 570 nm. The absorbance of the DMSO treated cells (under both conditions) was considered to be 100% and all samples were normalized to the DMSO treated cells. The statistical significance was calculated by Student's t-test.

## SUPPLEMENTARY FIGURES AND TABLES

















## Abbreviations

GBM: Glioblastoma
RBP: RNA binding protein
GSC: Glioma stem-like cell
NSC: Neural stem cell
DGC: Differentiated glioma cells
CNV: Copy Number Variation
TCGA: The Cancer Genome Atlas
REMBRANDT: Repository for Molecular Brain Neoplasia Data
miRNA: Micro RNA
lncRNA: Long noncoding RNA
WES: Whole Exome Sequencing
HR: Hazard Ratio
G-CIMP: Glioma-CpG island methylator phenotype
GSEA: Gene Set Enrichment Analysis
EMT: Epithelial-mesenchymal transition
