# Supplementary material for: Elucidation of the genetic and epigenetic landscape alterations in RNA binding proteins in glioblastoma
Source: Oncotarget. 2016 Dec 27;8(10):16650–68. doi: 10.18632/oncotarget.14287 (PMC5369992; doi:10.18632/oncotarget.14287)
Supplement: Supplementary file 1 [file oncotarget-08-16650-s001.pdf]

Elucidation of the genetic and epigenetic landscape alterations in RNA binding proteins in glioblastoma

SUPPLEMENTARY FIGURES AND TABLES

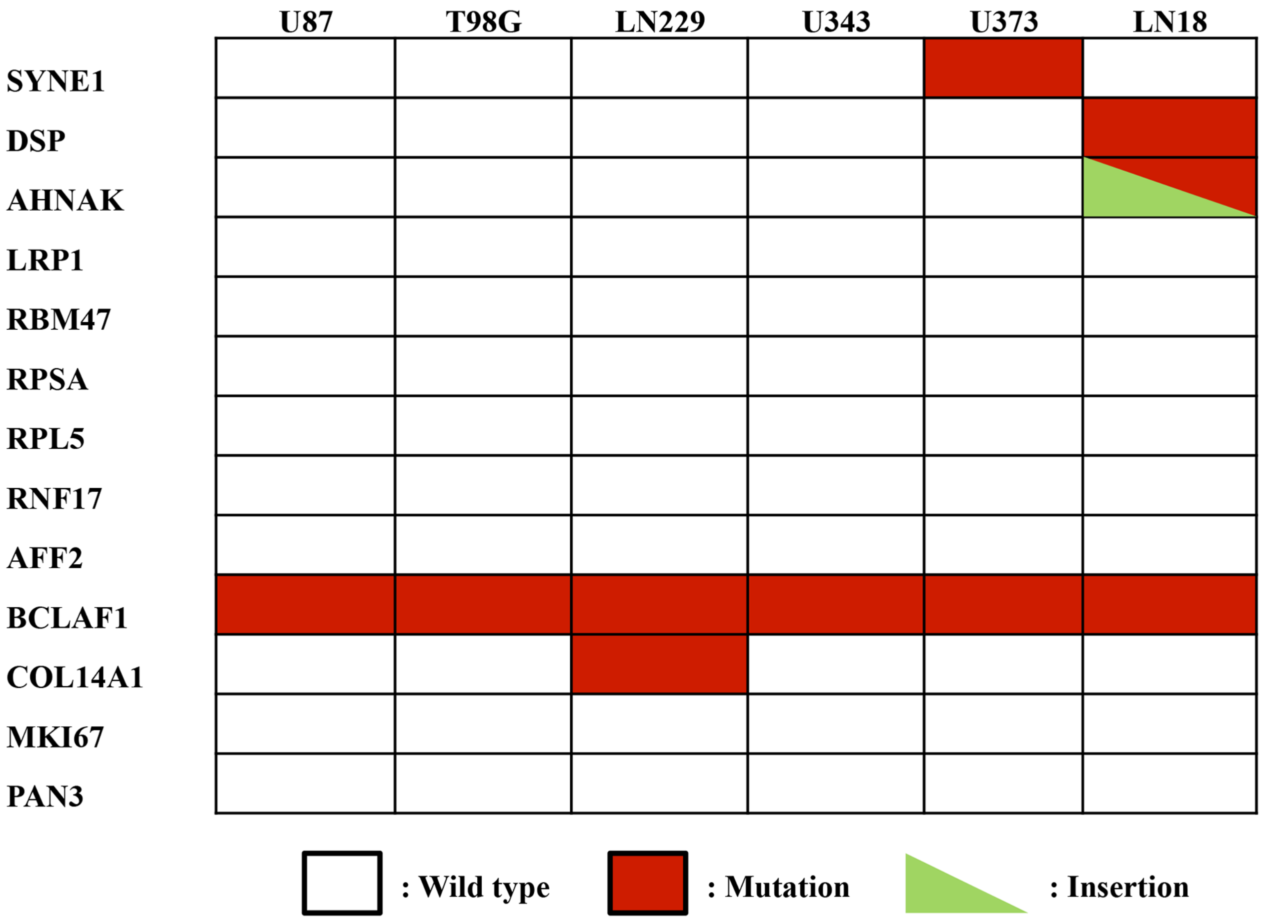

Supplementary Figure 1: Mutational status of 12 mutated RBPs in established GBM cell lines. The status of the mutated RBPs found in TCGA was checked in the data derived from the established GBM cell lines. The results are depicted graphically. Red boxes represent the cell lines where the RBP was mutated, while white boxes represent the cell lines where the RBP was wild type.

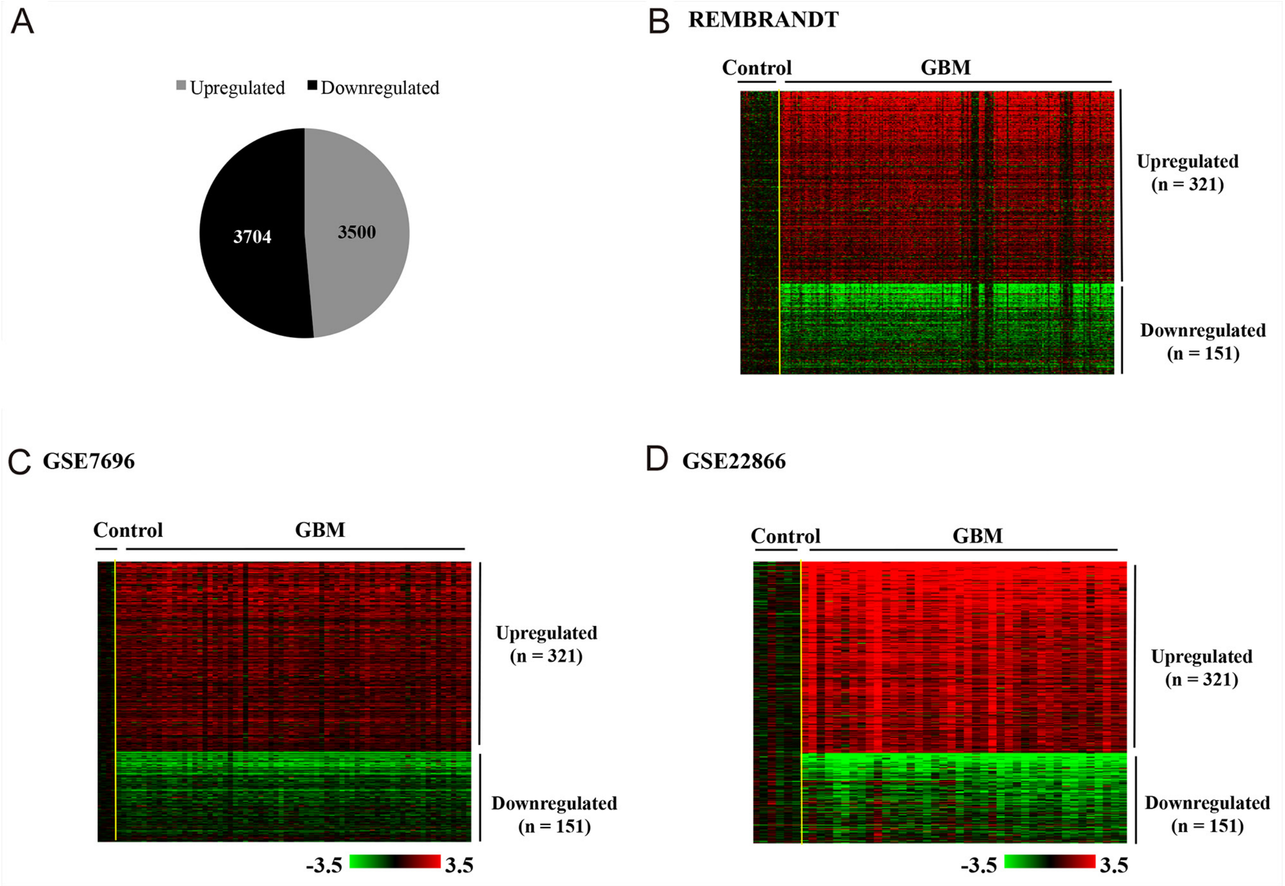

Supplementary Figure 2: Differentially regulated RBPs in GBM. **(A)** Total number of differentially regulated genes in GBM samples as compared to control samples (TCGA dataset is used). **(B, C, D)** Heat map representation of differentially regulated RBPs in REMBRANDT, GSE7696 and GSE22866 respectively.

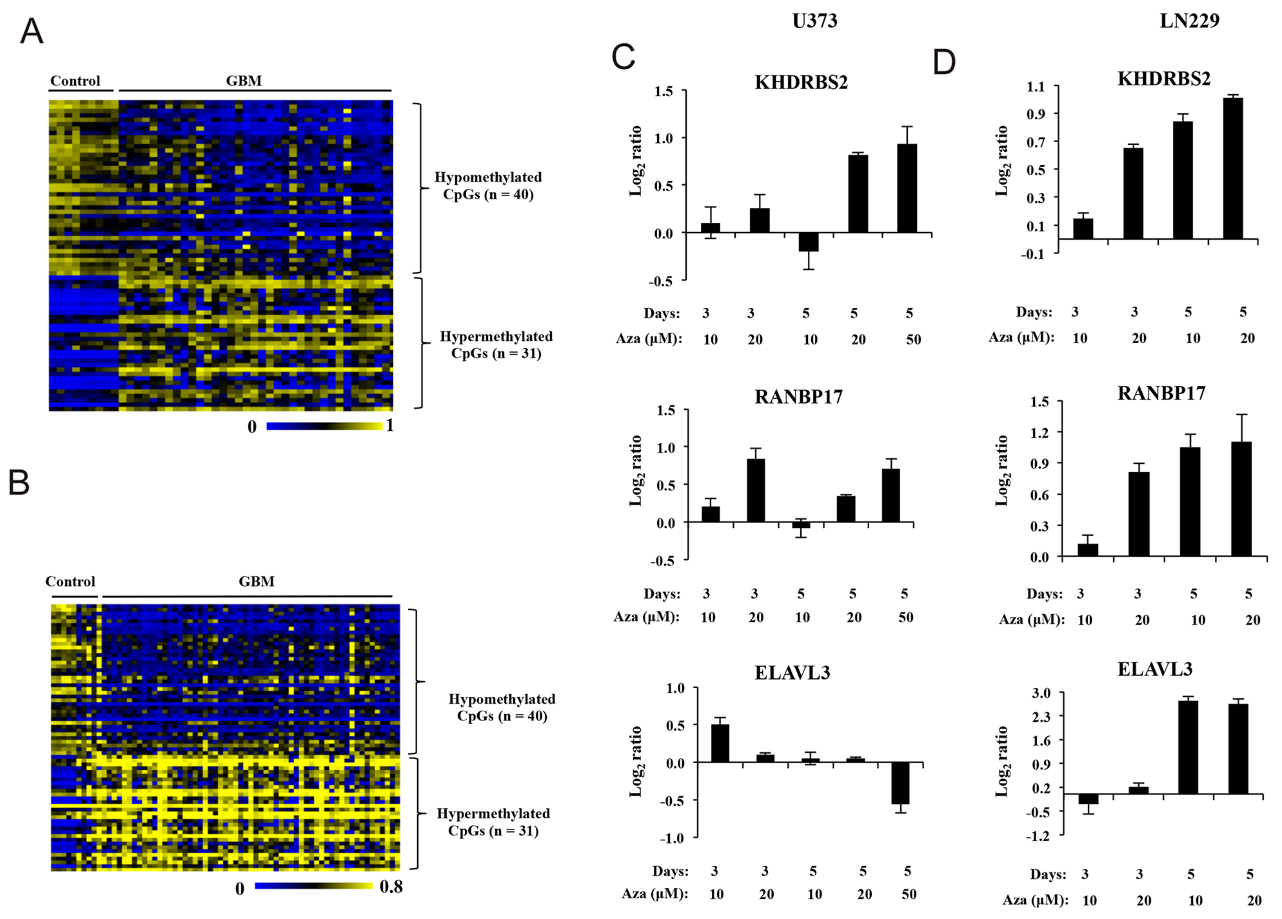

Supplementary Figure 3: Probable causes of differential expression of RBPs. (A) Heat map showing the methylation pattern of probes corresponding to differentially expressed RBPs that had differential methylation in GBM samples using GSE79122 for methylation analysis. **(B)** Heat map showing the methylation pattern of probes corresponding to differentially expressed RBPs that had differential methylation in GBM samples using GSE60274 for methylation analysis. **(C, D)** Transcript levels of KHDRBS2, RANBP17 and ELAVL3 (as assessed by qRT-PCR) in RNA was isolated from U373 **(C)** and LN229 **(D)** cells after treatment with 5-aza-2-deoxycytidine (DAC) for 3 and 5 days with 10, 20 and 50 μM concentrations.

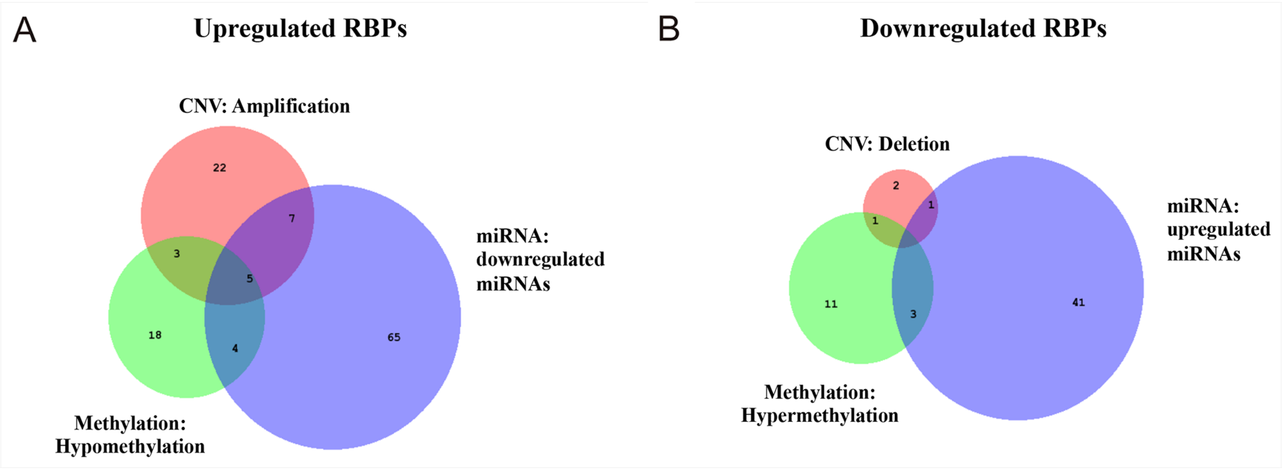

Supplementary Figure 4: Relative effect of the three factors analyzed on RBP gene expression. (A) Venn diagram showing the number of upregulated genes regulated by each of the parameter individually and in combination. **(B)** Venn diagram showing the number of downregulated genes regulated by each of the parameter individually and in combination.

Supplementary Table 1: List of RBPs catalogued and used in this study.

See Supplementary File 1

Supplementary Table 2A: Altered RBPs in TCGA GBM samples.

See Supplementary File 2

Supplementary Table 2B: Different types of genetic alterations in RBPs in TCGA GBM samples.

See Supplementary File 2

Supplementary Table 2C: Altered RBPs in glioma cell lines.

See Supplementary File 2

Supplementary Table 2D: Functional prediction of mutations found in RBPs.

See Supplementary File 2

Supplementary Table 2E: Reported function of wild type and mutated 13 most altered RBPs (in various cancers) as indicated in the mentioned references (given as pubmed IDs).

See Supplementary File 2

Supplementary Table 2F: Univariate cox regression analysis of top 13 altered RBPs in TCGA GBM samples.

See Supplementary File 2

Supplementary Table 3: Differentially regulated RBPs in different data sets.

See Supplementary File 3

Supplementary Table 4A: Differentially expressed RBPs which showed significant CNVs.

See Supplementary File 4

Supplementary Table 4B: Differentially expressed RBPs which showed differetial methylation status.

See Supplementary File 4

Supplementary Table 4C: Differentially expressed RBPs and the miRNAs predicted to target these RBPs.

See Supplementary File 4

Supplementary Table 5A: RBPs identified that may play a role in transformation of normal brain cells to low grade glioma.

See Supplementary File 5

Supplementary Table 5B: RBPs identified that may play a role in aggressiveness of GBM.

See Supplementary File 5

Supplementary Table 6: Differentially regulated RBPs in GSCs versus NSC and DGC along with their univariate values in GBM.

See Supplementary File 6

Supplementary Table 7: Differentially regulated genes between high risk and low risk GBM patients.

See Supplementary File 7
